# Study on the impact of rural public sports facilities and instructors on residents’ participation in sports activities in China

**DOI:** 10.3389/fpubh.2025.1475321

**Published:** 2025-02-26

**Authors:** Xiujin Guo, Xiangjun Yang, Sujie Mao

**Affiliations:** ^1^NanJing Sport Institute, Nanjing, JiangSu, China; ^2^Department of Physical Education, Taizhou University, Taizhou, Zhejiang, China; ^3^Graduate Department, Harbin University of Sport, Haibin, Heilongjiang, China

**Keywords:** rural sports activities, public sports facilities, sports activity participation, sports facility instructors, rural sports

## Abstract

**Objective:**

This study aims to explore how rural public sports facilities and their instructors influence the participation of rural residents in sports activities under the background of China’s rural revitalization strategy. The goal is to provide strategies for the effective use and management of rural sports facilities, thereby encouraging rural residents to actively participate in sports activities, improve their quality of life, and support comprehensive rural revitalization.

**Methods:**

A cross-sectional study design was used, employing a stratified sampling method to distribute questionnaires to 5,000 residents in the eastern, central, western, and southern regions of China. Data analysis was conducted using R4.1.3 software. The main research indicators included the funding sources of sports facilities and the composition of social sports instructors, while residents’ basic characteristics were considered secondary indicators for correlation, regression, and cross-analysis.

**Results:**

Analysis of 3,956 valid questionnaires revealed that increasing age led to a decrease in the frequency of sports activities (−0.098), while higher education levels increased activity frequency (0.097). Facilities provided by county sports bureaus significantly promoted participation in sports activities (*B =* 0.320, *p <* 0.001). Facilities donated by villagers or township enterprises and the sports lottery fund also effectively increased participation (*B =* 0.219, *p <* 0.001; *B =* 0.159, *p =* 0.011). Sports facility instructors, particularly urban residents and retirees, significantly positively impacted activity time, while the absence of instructors reduced residents’ participation in sports activities (*B =* −0.445, *p <* 0.001).

**Conclusion:**

Age and education level are negatively correlated with sports activity participation, while the source of public sports facilities, especially those provided by county sports bureaus, the sports lottery fund, and village committees, significantly enhance participation. The presence of social sports instructors significantly increases the time and frequency of residents’ sports activities. Policymakers need to focus on the construction and management of public sports facilities, develop and utilize diversified funding sources, and enhance the role of sports facility instructors. By providing professional guidance and organizing diverse sports activities, rural residents’ enthusiasm for participation can be effectively stimulated.

## 1 Introduction

China’s Rural Revitalization Plan aims to comprehensively enhance the quality of rural economic, social, cultural, and ecological environments (1–3). The revitalization of sports is crucial for rural prosperity, highlighting the growing importance of rural residents’ participation in sports activities (4). Engaging in sports is a vital component of maintaining a healthy lifestyle for rural residents, promoting physical and mental health, reducing disease risk (5), improving life quality and well-being, effectively lowering healthcare costs, and increasing labor productivity, thus providing health security for rural revitalization. It not only improves the physical health of rural residents but also strengthens community cohesion, promotes cultural vibrancy, and injects vitality into rural revitalization (6). However, according to the World Health Organization (WHO), insufficient physical activity is a common issue worldwide. Nordic countries, relying on substantial government investment and well-developed community services, have achieved balanced urban–rural sports participation. The United States and Canada have improved participation rates through policy support and community-driven models, yet challenges persist among low-income groups. In contrast, middle- and low-income countries such as India and Brazil face low rural sports participation rates due to inadequate facilities and a lack of cultural awareness.Approximately 1.4 billion adults (27.5% of the global adult population) have physical activity levels below WHO standards (7, 8), which recommend at least 150 min of moderate-intensity physical activity per week. This issue is particularly severe in low- and middle-income countries, due to rapid urbanization, lifestyle changes, and insufficient public facilities for physical activity (9).

According to the 2020 *Survey Bulletin on the Status of National Fitness Activities*, the proportion of rural residents in China who regularly engage in physical exercise is only 10.4%, significantly lower than the 19.5% among urban residents. Although the national per capita area of sports venues has reached 2.89 square meters, exceeding the target set in the *14th Five-Year Plan for Sports Development*, issues such as inadequate supply of sports facilities, low economic levels, weak awareness of fitness, and lack of basic infrastructure in rural areas severely restrict rural residents’ participation in sports activities. This low participation rate not only affects physical health and quality of life but also hinders the implementation of the rural revitalization strategy. Enhancing the participation rate in sports activities among rural residents is essential for strengthening community cohesion and improving health outcomes.

Public sports facilities play a critical role in promoting sports activities among rural residents (10, 11). They serve not only as venues for physical exercise but also as platforms for community interaction and cohesion. These facilities include sports fields, fitness equipment, and open activity spaces, which in rural areas are often represented by simple basketball courts, tracks, or open spaces for collective activities. However, despite the national per capita area of sports venues reaching 2.89 square meters, with a total of 4.5927 million facilities, the supply and quality of sports facilities in rural areas remain significantly lower than in urban areas. Many villages lack basic facilities, or existing facilities suffer from inadequate maintenance. Challenges such as insufficient funding, weak management, and limited promotion of activities further constrain residents’ participation in sports (12). Optimizing and improving rural public sports facilities is crucial not only for increasing residents’ participation in sports and enhancing their quality of life but also for injecting vitality into rural revitalization by organizing diversified sports activities and fostering community interaction.

Previous studies have mainly focused on the quantity of rural sports facilities, construction models, and rural-specific sports projects, providing valuable insights into the basic status of rural sports facilities (13–15). However, there is a lack of in-depth analysis and discussion on the funding sources of sports facilities and the role of sports instructors in promoting residents’ participation in sports activities. Existing studies predominantly focus on the quantity and construction models of rural sports facilities, lacking exploration of the synergistic effects between facilities and instructors. This study, adopting a multidisciplinary perspective, emphasizes the dual role of funding sources and instructor functions, filling a theoretical gap and providing systematic support for policy formulation.

In light of this, this study analyzes the funding sources of rural sports facilities and the organization and role of public sports facility instructors from sociological, economic, and multidisciplinary perspectives to explore the impact of rural sports facilities on promoting residents’ participation in sports activities. By examining the funding support models, management and maintenance strategies of sports facilities, and the actual effectiveness of sports instructors in guiding and motivating rural residents, this research aims to provide policymakers, community planners, and sports activity organizers with more comprehensive and in-depth insights and recommendations. This will promote the more efficient use and management of rural sports facilities, stimulate residents’ enthusiasm for sports activities, and improve their quality of life.

## 2 Research methods

This study employs a cross-sectional design and is completed according to the STROBE checklist (16). It aims to thoroughly investigate the participation of rural residents in sports activities in sub-county regions of China. The study examines the sources of public sports facilities and the impact of social sports instructors on residents’ participation in sports activities, providing empirical support for developing more effective sports policies and enhancing rural sports participation.

### 2.1 Study setting

This study is designed to deeply analyze the participation of rural residents in sports activities in China, using a stratified sampling method to conduct surveys in specific areas. Based on the “Method of Division of Eastern, Central, Western, and Northeastern Regions” published by the National Bureau of Statistics of China (17), the study regions are subdivided into four geographic layers: Eastern, Central, Western, and Northeastern. The selected regions include Guangdong, Zhejiang, and Jiangsu (Eastern Region); Anhui, Henan, and Hunan (Central Region); Chongqing, Shaanxi, and Guizhou (Western Region); and Heilongjiang Province (Northeastern Region).To ensure the representativeness of the study regions, provinces with median GDP values were selected to represent varying levels of economic development. The selection achieved balanced coverage across the eastern, central, western, and northeastern regions of China, prioritizing areas with a higher proportion of rural populations and diverse levels of sports facility accessibility. This approach provides a scientific basis for analyzing the relationship between rural public sports facilities and residents’ participation in sports activities.

In each selected province, two prefecture-level cities were chosen based on the median GDP, each prefecture-level city further selected two counties (cities or districts), and each county (city or district) selected two townships. In each township, two representative villages were selected for field surveys. This study utilized a questionnaire (approved by the expert panel of Shanghai University of Sport), targeting 5,000 rural residents. The stratified sampling method ensures the broad representativeness and deep coverage of the study results, while also considering the differences in economic development, cultural traditions, and sports activity participation across different regions of China. The questionnaire distribution period was from March to August 2023, and the recovery period was from September to October 2023. The aim was to comprehensively understand the level of participation in sports activities among rural residents and the roles of public sports facility sources and social sports instructors in promoting participation in sports activities.

### 2.2 Participants

#### 2.2.1 Inclusion criteria

Residents living in the selected rural areas of China, aged 18 and above, able to understand the questionnaire content, and willing to participate in the study.

#### 2.2.2 Exclusion criteria

Minors, residents not living in the designated study areas, and individuals unable to understand or complete the questionnaire due to health or cognitive issues.

#### 2.2.3 Recruitment process

A stratified sampling method was used to recruit participants, ensuring broad representativeness of the sample. First, based on the regional divisions by the National Bureau of Statistics, two prefecture-level cities were selected based on the median GDP; within each prefecture-level city, two counties (cities or districts) were selected, and each county further selected two townships, with each township ultimately selecting two representative villages. Invitations to participate were extended to all residents meeting the inclusion criteria through the assistance and announcements of village committees.

### 2.3 Sample size

The sample size determination was based on the estimated effect size from previous studies and the anticipated data analysis methods. Considering the cross-sectional study design, the expected minimum effect size, an alpha level of 0.05, and a statistical power of 0.80, the sample size was calculated using GPower software, resulting in a required sample of 3,571 individuals. To account for a potential 30% data loss and non-response rate, the final total sample size was set at 5,000 individuals, ensuring sufficient statistical power to detect the effects hypothesized in the study.

To comprehensively assess the impact of rural public sports facilities on residents’ participation in sports activities, the study employed a comprehensive research method, primarily including an investigation of the basic characteristics of rural residents and an in-depth exploration of their sports activity participation.

### 2.4 Measurement indicators

1. Basic Information of Rural Residents: The study will collect basic information on rural residents, including age, gender, education, and income.
2. Participation Time and Frequency of Sports Activities: Detailed records of the average duration and frequency of rural residents’ participation in sports activities. These data will directly reflect the actual participation in sports activities among rural residents, providing direct evidence for assessing sports activity behavior.
3. The questionnaire for this study was distributed with the assistance of village committees and completed independently by participants. To ensure the independence and authenticity of responses, the research team provided detailed instructions during the distribution process and implemented an anonymous response mechanism. The questionnaire design was reviewed by an expert panel from the Shanghai University of Sport and underwent a small-scale pilot test prior to implementation. Fifty rural residents from different regions were selected to evaluate the readability, logic, and practicality of the questionnaire. The pilot test results indicated that the questionnaire items clearly reflected the research objectives, and adjustments were made to certain items based on feedback. After the questionnaires were collected, a double-checking mechanism was employed during data entry and cleaning to eliminate invalid or illogical responses, ensuring the validity and representativeness of the data. This approach not only ensured the scientific rigor of the questionnaire but also enhanced the reliability of the data.
4. Sources of Public Sports Facilities: The funding sources of rural public sports facilities, including government investment, social donations, corporate sponsorships, and other forms.
5. Composition of Public Sports Facility Instructors: Analysis of the qualifications, training background, and working methods of rural sports facility instructors, and how they influence rural residents’ participation in sports activities.

By analyzing the above indicators, the study will explore the actual impact of rural public sports facilities and instructors on promoting residents’ participation in sports activities, providing scientific strategies and suggestions for promoting and developing rural sports activities.

### 2.5 Data analysis

Data from the questionnaires will be entered into EXCEL and analyzed using R software version 4.3.1. Statistical analyses will include descriptive analysis, correlation analysis, regression analysis, and cross-analysis. Descriptive statistics will cover demographic information and an overview of sports activity participation, involving calculations of means, standard deviations, frequencies, and percentages. Correlation analysis will determine the strength and direction of associations between study variables, using Pearson correlation coefficients to assess statistical significance. Regression analysis will evaluate the relationship between rural residents’ participation in sports activities and potential predictor variables, including basic characteristics (age, education, occupation, etc.), sources of public sports facilities, and the impact of social sports instructors. A multiple linear regression model will be constructed to quantitatively assess the contribution of each independent variable to sports activity participation while controlling for potential confounding variables. Cross-analysis will be applied to investigate differences in the frequency and duration of sports activity participation based on the sources of public sports facilities.

To ensure robust analysis, the study included age, gender, education level, and income as covariates in the regression models. These variables, while not the primary research objectives, were included to control for their potential confounding effects on the relationship between public sports facilities, social sports instructors, and rural residents’ participation in sports activities.

## 3 Results

### 3.1 Sample characteristics

To ensure sufficient sample size, this study distributed a total of 5,000 questionnaires to residents in rural areas of China, successfully retrieving 4,625 questionnaires. Due to incomplete responses in 309 questionnaires and 3,956 respondents not meeting the criteria of postmenopausal women, these data were excluded from the final analysis. Ultimately, 3,956 complete and usable questionnaires were included in the study.

#### 3.1.1 Basic information of subjects

Survey data indicated regional differences in participant distribution, with the Northeast region having the highest proportion (31.54%), while the Eastern, Central, and Western regions were relatively balanced. Regarding gender, females slightly outnumbered males, accounting for 50.21%. The age distribution was mainly between 31 and 59 years. In terms of educational level, most rural residents had junior high school education or below, comprising over half of the sample. Participation levels varied significantly among different occupational groups, with village and township officials having relatively high proportions (32.49 and 24.06%, respectively), while farmers, migrant workers, and teachers had lower proportions. Economic status showed income disparities, with higher proportions of incomes below 5,000 yuan and between 5,000–9,999 yuan (12.77 and 16.08%, respectively), which might affect the demand for and participation in sports facility services.

#### 3.1.2 Level of sports activity participation among rural residents

**Figure 1:**
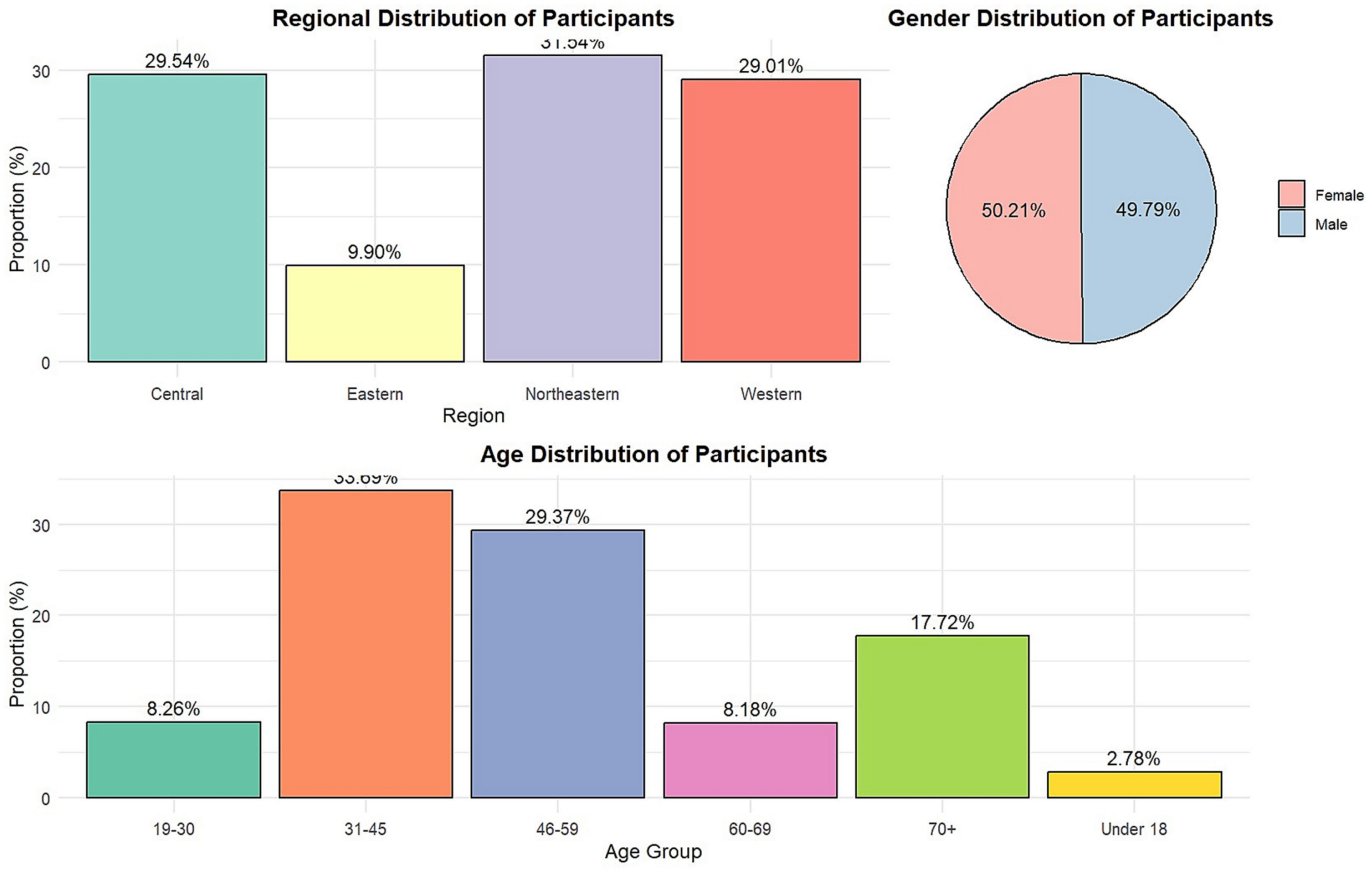
Demographics and regional distribution of participants.

The results showed significant diversity in the weekly frequency of sports activities among participants. Among 3,595 individuals surveyed, 38.19% chose “not fixed, occasionally participate” as their main frequency of sports activity. In contrast, 20.81% of participants engaged in activities more than three times per week, 20.56% participated 1–3 times per week, and 20.45% did not engage in sports activities, highlighting significant differences in participation levels and reflecting the individualized needs and diverse habits of rural residents in sports activities. Among the 20.45% of individuals who did not participate in sports activities, several notable characteristics were identified: 25.90% were aged 60 and above, with declining physical function potentially limiting participation; 8.62% were farmers, and 12.66% were semi-employed, where high labor intensity and life pressures reduced available time for activities; 28.85% belonged to low-income groups (annual income below 10,000 RMB), with economic constraints limiting access to opportunities; 52.63% had lower education levels, reflecting insufficient health awareness and knowledge about sports; and non-participation rates were higher in the northeastern (31.54%) and western (29.01%) regions, highlighting the impact of insufficient public sports facilities on participation. Additionally, the results demonstrated diversity in the duration of each sports activity session. Among the participants, 41.00% chose activity times exceeding 90 min, 40.03% chose a range of 30–60 min, 21.97% had activity times less than 30 min, and 11.71% selected durations of 61–90 min (Table 1; Figure 1).

**Table 1:** Demographics, occupations, income levels, and regional distribution of participants in the rural sports study.

Category	Count	Proportion	Age group	Count	Proportion
Female	1,805	50.21%	60–69 years old	294	8.18%
Male	1,790	49.79%	46–59 years old	1,056	29.37%
Job Type	Count	Proportion	31–45 years old	1,211	33.69%
Migrant Worker	105	2.92%	70+ years old	637	17.72%
Farmer	310	8.62%	19–30 years old	297	8.26%
Village Cadre	1,168	32.49%	Under 18	100	2.78%
Township Cadre	865	24.06%	Region	Count	Proportion
Self-employed	514	14.30%	Eastern	356	9.90%
Semi-employed	455	12.66%	Northeastern	1,134	31.54%
Teacher	165	4.59%	Central	1,062	29.54%
Others	13	0.36%	Western	1,043	29.01%
Income Level	Count	Proportion	Education Level	Count	Proportion
< 5,000 RMB	459	12.77%	High school/technical school	493	13.71%
5,000–9,999 RMB	578	16.08%	Junior high school	1,128	31.38%
10,000–14,999 RMB	506	14.08%	Primary school or below	764	21.25%
30,000–49,999 RMB	360	10.01%	Associate degree	369	10.26%
20,000–29,999 RMB	375	10.43%	Bachelor’s degree	759	21.11%
50,000–99,999 RMB	506	14.08%	Graduate degree	82	2.28%
15,000–19,999 RMB	454	12.63%			
100,000+ RMB	357	9.93%			

### 3.2 Correlation analysis of sports activity participation levels and characteristics of rural residents

Correlation analysis between the basic characteristics of rural residents and their sports activity behaviors (Table 2) revealed that the relationship between sports activity frequency and individual characteristics was relatively weak. The correlation coefficients for region, gender, age, education, occupation, and household income were close to zero, indicating no significant linear relationship between these characteristics and sports activity frequency. However, age and education level showed a significant negative correlation with sports activity frequency, meaning that as age and education level increased, the frequency of sports activities decreased. Conversely, the relationship between sports activity time and individual characteristics was more complex. Correlation coefficients for region and gender were small, whereas those for age, education, occupation, and household income were more significant. Table 2 shows that sports activity time negatively correlated with age and education level and positively correlated with occupation and household income. This suggests that as age and education level increase, sports activity time may decrease, while higher household income is associated with increased participation in sports activities.

**Table 2:** Correlation between basic characteristics and frequency/time of participation in sports activities.

Region	Gender	Age	Education	Occupation	Household income	Region
Frequency of participation in sports activities	−0.032	0.019	−0.124**	0.146**	0.047**	0.094**
Time per participation in sports activities	−0.198**	0.007	−0.103**	0.260**	0.079**	0.091**

*p < 0.05; **p < 0.01.

### 3.3 Regression analysis of rural residents’ characteristics and sports activity levels

Regression analysis of basic characteristics and sports behaviors (Table 3) revealed that the intercept of the regression equation was 1.624, indicating that, with other variables held constant, the basic participation frequency in sports activities was 1.624 (*p* < 0.01), which is statistically significant, with a 95% CI of 1.359–1.889. The unstandardized coefficients for region, gender, age, education, occupation, and household income were − 0.023, 0.035, −0.097, 0.079, 0.015, and 0.034, respectively. The standardized coefficients (Beta) represent the impact of a one-unit standard deviation change in the independent variables on the dependent variable. Significance tests showed that age and education level significantly affected sports activity frequency (*p* < 0.01), while other variables did not have a significant impact. The results indicate a negative correlation between age and sports activity frequency, with a one-standard-deviation increase in age leading to a decrease in activity frequency by 0.098. Conversely, education level positively correlated with sports activity frequency, with a one-standard-deviation increase in education leading to an increase in activity frequency by 0.097. Thus, as age decreases and education level increases, the frequency of participation in sports activities among rural residents increases.

**Table 3:** Regression analysis of basic characteristics and frequency of participation in sports activities.

Regression coefficients (Intermediate process; *n* = 3,595)							
	Unstandardized Coefficients		Standardized Coefficients	*t*	*p*	95% CI	VIF
	*B*	Standard error	*Beta*				
Constant	1.624	0.135	–	12.022	0.000**	1.359–1.889	–
Region	−0.023	0.020	−0.020	−1.153	0.249	−0.063–0.016	1.074
Gender	0.035	0.038	0.015	0.914	0.361	−0.040–0.110	1.014
Age	−0.097	0.017	−0.098	−5.582	0.000**	−0.131–−0.063	1.139
Education	0.079	0.015	0.097	5.381	0.000**	0.050–0.108	1.198
Occupation	0.015	0.014	0.018	1.083	0.279	−0.012–0.042	1.051
Household income	0.034	0.009	0.068	3.947	0.000**	0.017–0.052	1.095

*p < 0.05; **p < 0.01. Dependent variable: Frequency of participation in sports activities.

Regression analysis of the sports activity time revealed the impact of region, gender, age, education, occupation, and household income on the frequency of participation (Table 4). Region (*β* = −0.173, *p <* 0.01) and age (*β* = −0.062, *p <* 0.01) had significant negative effects on sports activity frequency, while education level (*β* = 0.210, *p <* 0.01) had a significant positive impact. Occupation (*β* = 0.035, *p <* 0.05) also positively affected sports activity frequency, although the impact was relatively small. In contrast, the effects of gender (*p =* 0.367) and household income (*p =* 0.336) were not statistically significant. The 95% CI and VIF values further enhanced the explanatory power and reliability of the analysis results.

**Table 4:** Regression analysis of basic characteristics and time per participation in sports activities.

Regression coefficients (Intermediate process; *n* = 3,595)								
		Unstandardized Coefficients		Standardized Coefficients	*t*	*p*	95% CI	VIF
		*B*	Standard error	*Beta*				
Constant	1.748	0.125	–	14.007	0.000**	1.504 ~ 1.993	–	
Region	−0.197	0.019	−0.173	−10.536	0.000**	−0.234 ~ −0.161	1.074	
Gender	−0.032	0.036	−0.014	−0.902	0.367	−0.102 ~ 0.038	1.014	
Age	−0.058	0.016	−0.062	−3.646	0.000**	−0.090 ~ −0.027	1.139	
Education	0.165	0.014	0.210	12.124	0.000**	0.138 ~ 0.191	1.198	
Occupation	0.027	0.013	0.035	2.158	0.031*	0.003 ~ 0.052	1.051	
Household Income	0.008	0.008	0.016	0.963	0.336	−0.008 ~ 0.024	1.095	

*p < 0.05; **p < 0.01. Dependent variable: Time per participation in sports activities.

The study results indicated that as age increases, sports activity frequency decreases, while higher education levels promote participation in sports activities. Although other factors, such as region, gender, occupation, and household income, were considered, their impact on sports activity frequency did not reach statistical significance. These findings emphasize the importance of age and education level in promoting participation in sports activities, providing essential bases for designing targeted sports promotion strategies.

### 3.4 Cross-analysis of sources of public sports facilities and sports activity levels of rural residents

The study conducted regression and cross-analysis to explore the relationship between the sources of public sports facilities and the frequency and duration of rural residents’ participation in sports activities. The sources of public sports facilities included purchases by village committees, provision by township governments, donations from village enterprises, donations from the sports lottery fund, provision by county sports bureaus, donations from urban residents or enterprises, and others.

Regression analysis results of public sports facilities and rural residents’ sports activity levels showed that facilities provided by county sports bureaus had the most significant positive impact on sports activity frequency (*B =* 0.320, *p <* 0.001), indicating that such facilities significantly increased the frequency of sports activity participation. Facilities donated by villagers or township enterprises (*B =* 0.219, *p <* 0.001) and the sports lottery fund (*B =* 0.159, *p =* 0.011) also had a significant positive impact on activity frequency. In contrast, facilities from other sources had a slight negative impact on activity frequency, although this result was not statistically significant (*B =* −0.089, *p =* 0.076). Collinearity diagnostics were assessed using the variance inflation factor (VIF) and tolerance indicators, showing that all variables had VIF values below 10 and tolerance values above 0.1, indicating no severe collinearity problems in the model.

**Figure 2:**
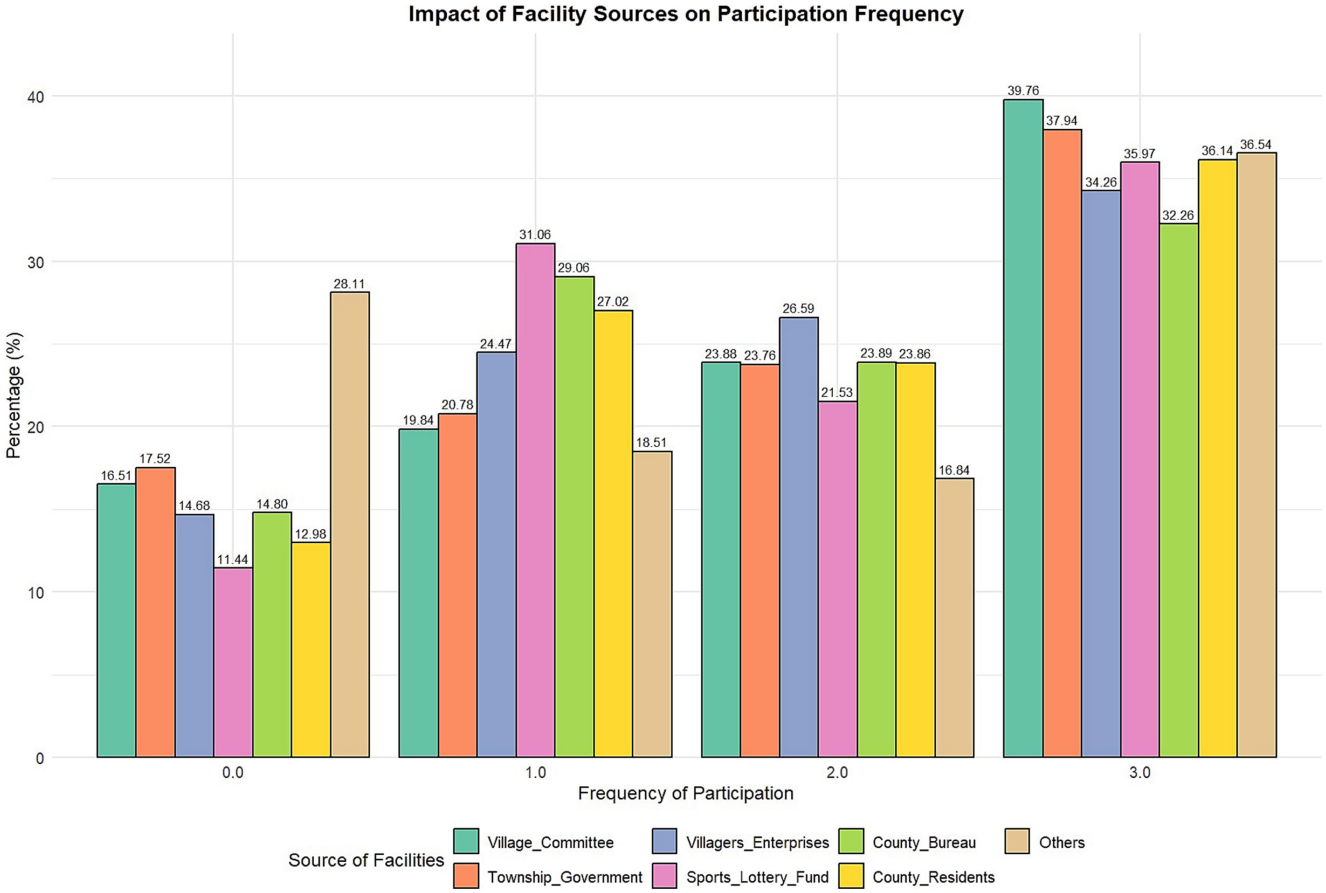
Frequency of participation by source of sports facilities.

**Figure 3:**
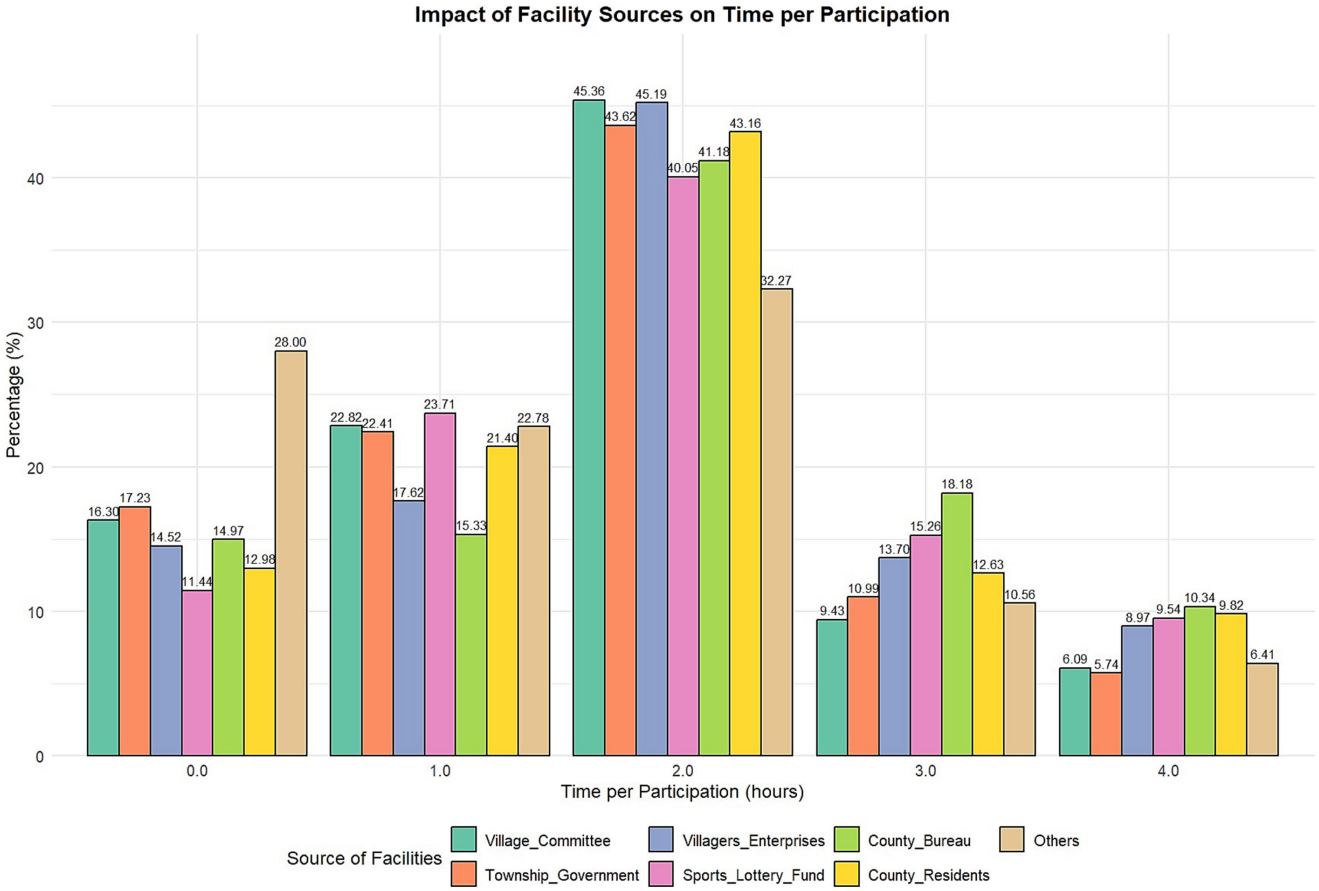
Time per participation by source of sports facilities.

Cross-analysis of the frequency of sports activity participation among rural residents (Table 5; Figure 2) revealed a significant relationship between public sports facilities and activity frequency (χ^2^ = 136.099, *p <* 0.001), indicating the critical role of facility sources in promoting sports activity participation (Table 6; Figure 3). Facilities donated by the sports lottery fund showed a significant positive correlation with higher frequency (three or more times) of sports activity participation, with 31.06% of individuals participating in sports activities three or more times. Additionally, facilities purchased by village committees and provided by township governments positively impacted sports activity participation, particularly in the frequency category of three or more times, accounting for 39.76 and 37.94%, respectively. This demonstrates the significant role of the government in providing sports facilities. Facilities donated by villagers or township enterprises also had a notable association with higher participation frequencies (34.26% of individuals participated in sports activities three or more times), highlighting the importance of community and private sector contributions in promoting sports activities.

**Table 5:** Cross-analysis of sources of public sports facilities and frequency of sports activities.

Sources of sports facilities and frequency of participation in sports activities—cross tabulation (%)								
Title	Item	Purchased by Village committee	Provided by township government	Donated by villagers or township enterprises	Donated by sports lottery fund	Provided by county sports bureau	Donated by county residents or enterprises	Others
Frequency of participation in sports activities	0.0	233(16.51)	247(17.52)	90(14.68)	42(11.44)	83(14.80)	37(12.98)	237(28.11)
	1.0	280(19.84)	293(20.78)	150(24.47)	114(31.06)	163(29.06)	77(27.02)	156(18.51)
	2.0	337(23.88)	335(23.76)	163(26.59)	79(21.53)	134(23.89)	68(23.86)	142(16.84)
	3.0	561(39.76)	535(37.94)	210(34.26)	132(35.97)	181(32.26)	103(36.14)	308(36.54)

Chi-Square Test: χ^2^ = 136.099, *p* = 0.000.

**Table 6:** Cross-analysis of sources of public sports facilities and time per participation in sports activities.

Sources of sports facilities and time per participation in sports activities—cross tabulation (%)								
Title	Item	Purchased by village committee	Provided by township government	Donated by villagers or township enterprises	Donated by sports lottery fund	Provided by county sports bureau	Donated by county residents or enterprises	Others
Time per participation in sports activities	0.0	230(16.30)	243(17.23)	89(14.52)	42(11.44)	84(14.97)	37(12.98)	236(28.00)
	1.0	322(22.82)	316(22.41)	108(17.62)	87(23.71)	86(15.33)	61(21.40)	192(22.78)
	2.0	640(45.36)	615(43.62)	277(45.19)	147(40.05)	231(41.18)	123(43.16)	272(32.27)
	3.0	133(9.43)	155(10.99)	84(13.70)	56(15.26)	102(18.18)	36(12.63)	89(10.56)
	4.0	86(6.09)	81(5.74)	55(8.97)	35(9.54)	58(10.34)	28(9.82)	54(6.41)

Chi-Square Test: χ^2^ = 169.913, *p* = 0.000.

The sources of public sports facilities have a significant correlation with the level of sports activity participation among rural residents. Facilities donated by the Sports Lottery Fund, provided by township governments, and purchased by village committees significantly promoted residents’ participation in sports activities, especially when participation frequency was high. There is a notable positive correlation between facilities donated by the Sports Lottery Fund and participation in activities three times or more, highlighting the importance of financial support in promoting rural sports activities. Additionally, contributions at the government and community levels play a crucial role in enhancing sports activity participation. Diversified sources of sports facilities are essential for stimulating rural residents’ participation in sports activities.

### 3.5 The impact of public social sports instructors on rural residents’ participation in sports activities

Linear regression analysis was used to explore the relationship between the composition of rural social sports instructors and their impact on the time and frequency of participation in sports activities. This analysis covered 3,595 respondents and revealed the influence of instructors on the time and frequency of sports activities. The analysis included villagers with or without migrant work experience, retired urban residents who returned to the countryside, village officials or volunteers on temporary assignments, urban residents working in villages, and sports teachers or township officials living in rural areas.

The regression analysis results of residents’ sports activity time (Table 7) showed that facility instructors who were urban residents working in the village had the most significant positive impact on sports activity time (*B =* 0.265, *p <* 0.001). Retired urban residents who returned to the countryside as facility instructors also showed a positive contribution to sports activity time (*B =* 0.245, *p <* 0.001). Conversely, the absence of social sports instructors (*B =* −0.445, *p <* 0.001) and uncertainty about the existence of sports organizations (*B =* −0.146, *p =* 0.011) had a significant negative impact on sports activity time, indicating the crucial role of facility instructors in promoting residents’ sports activities. Although the overall explanatory power of the model was limited (Adjusted R^2^ = 0.093), the F-test [*F* (18,3,576) = 21.411, *p <* 0.000] confirmed the statistical significance of the model.

**Table 7:** Regression analysis of the composition of rural social sports instructors and the frequency of participation in sports activities.

Results of linear regression analysis (*n* = 3,595)							
	Unstandardized coefficients		Standardized coefficients	*t*	*p*	Collinearity diagnostics	
	*B*	Standard error	*Beta*			VIF	Tolerance
Constant	1.646	0.051	–	32.338	0.000**	-	–
Villagers with migrant work experience	−0.055	0.064	−0.018	−0.861	0.389	1.649	0.607
Retired urban residents returned to countryside	0.021	0.063	0.006	0.326	0.745	1.565	0.639
Villagers without migrant work experience	0.122	0.071	0.031	1.735	0.083	1.305	0.766
Temporary village officials or volunteers	−0.039	0.061	−0.012	−0.633	0.527	1.511	0.662
Urban residents working in villages	0.265	0.071	0.067	3.725	0.000**	1.300	0.769
Rural sports teachers or township cadres living in rural areas	0.099	0.066	0.027	1.502	0.133	1.286	0.777
Other	0.062	0.075	0.016	0.826	0.409	1.410	0.709
Unknown	−0.146	0.057	−0.064	−2.541	0.011*	2.516	0.397
No sports organization	−0.445	0.066	−0.142	−6.748	0.000**	1.744	0.573
Villagers with migrant work experience	0.080	0.068	0.024	1.187	0.235	1.672	0.598
Retired urban residents returned to countryside	0.245	0.064	0.077	3.846	0.000**	1.602	0.624
Villagers without migrant work experience	0.079	0.075	0.020	1.054	0.292	1.358	0.736
Temporary village officials or volunteers	−0.123	0.065	−0.037	−1.898	0.058	1.480	0.676
Urban residents working in villages	0.315	0.072	0.080	4.366	0.000**	1.316	0.760
Rural sports teachers or township cadres living in rural areas	0.163	0.066	0.046	2.477	0.013*	1.357	0.737
Others	0.206	0.081	0.049	2.547	0.011*	1.484	0.674
No social sports instructors	0.059	0.064	0.019	0.921	0.357	1.665	0.600
Unknown	−0.128	0.059	−0.057	−2.179	0.029*	2.716	0.368
*R* ^2^	0.097						
Adjusted R^2^	0.093						
*F*	*F* (18,3,576) = 21.411, *p* = 0.000						
D-W value	1.566						

Dependent variable: Time per participation in sports activities. *p < 0.05; **p < 0.01.

The analysis of sports activity frequency (Table 8) showed that most variables’ unstandardized coefficients (B) and standardized coefficients (Beta) indicated that the characteristics of these sports facility instructors did not have a statistically significant impact on the frequency of sports activities. The regression analysis indicated that in the absence of social sports instructors, the frequency of sports activity participation significantly decreased (*B =* −0.304, *p <* 0.001), suggesting that the presence of sports organizations is crucial for increasing the frequency of sports activities. Retired urban residents who returned to the countryside (*B =* 0.187, *p =* 0.007) and uncertainty about the existence of social sports instructors (*B =* −0.179, *p =* 0.005) also had a significant impact on sports activity frequency.

**Table 8:** Regression analysis of the composition of rural social sports instructors and the frequency of participation in sports activities.

Results of Linear Regression Analysis (*n* = 3,595)							
	Unstandardized Coefficients		Standardized Coefficients	*t*	*p*	Collinearity Diagnostics	
	B	Standard error	*Beta*			VIF	Tolerance
Constant	1.796	0.055	–	32.508	0.000**	–	–
Villagers with migrant work experience	−0.099	0.070	−0.030	−1.429	0.153	1.649	0.607
Retired urban residents returned to countryside	−0.001	0.069	−0.000	−0.019	0.985	1.565	0.639
Villagers without migrant work experience	0.031	0.077	0.008	0.405	0.685	1.305	0.766
Temporary village officials or volunteers	0.076	0.067	0.023	1.133	0.257	1.511	0.662
Urban residents working in villages	−0.045	0.077	−0.011	−0.588	0.557	1.300	0.769
Rural sports teachers or township cadres living in rural areas	−0.036	0.072	−0.009	−0.502	0.616	1.286	0.777
Other	0.146	0.081	0.035	1.788	0.074	1.410	0.709
Unknown	0.127	0.062	0.054	2.045	0.041*	2.516	0.397
No sports organization	−0.304	0.072	−0.092	−4.244	0.000**	1.744	0.573
Villagers with migrant work experience	−0.076	0.073	−0.022	−1.039	0.299	1.672	0.598
Retired urban residents returned to countryside	0.187	0.069	0.056	2.701	0.007**	1.602	0.624
Villagers without migrant work experience	0.048	0.082	0.011	0.589	0.556	1.358	0.736
Temporary village officials or volunteers	0.024	0.070	0.007	0.337	0.736	1.480	0.676
Urban residents working in villages	−0.124	0.078	−0.030	−1.582	0.114	1.316	0.760
Rural sports teachers or township cadres living in rural areas	0.029	0.071	0.008	0.401	0.688	1.357	0.737
Others	−0.017	0.088	−0.004	−0.193	0.847	1.484	0.674
No social sports instructors	0.144	0.070	0.044	2.069	0.039*	1.665	0.600
Unknown	−0.179	0.064	−0.076	−2.797	0.005**	2.716	0.368
*R* ^2^	0.026						
Adjusted R^2^	0.021						
*F*	*F* (18,3,576) = 5.372, *p* = 0.000						
D-W value	1.672						

Dependent variable: Frequency of participation in sports activities. *p < 0.05; **p < 0.01.

Linear regression analysis revealed the significant impact of sports facility instructors on residents’ sports activity time and frequency. Urban residents working in the village and retired urban residents who returned to the countryside as social sports instructors had a notable positive contribution to sports activity time, while the absence of social sports instructors significantly reduced both the time and frequency of participation in sports activities. These results emphasize the importance of sports organizations and facility instructors in promoting participation in sports activities. The F-test confirmed the statistical significance of the analysis, indicating that enhancing guidance and support for sports facilities is necessary to increase villagers’ participation in sports activities.

## 4 Discussion

### 4.1 The impact of age and education level of rural residents on participation in sports activities

In the context of China’s rural revitalization, enhancing the participation of rural residents in sports activities is of great significance for residents’ health and rural development. This study explored the impact of rural public sports facilities on residents’ participation in sports activities. The research found that age and education level are negatively correlated with participation in sports activities, while the sources of public sports facilities, especially those provided by county sports bureaus, donated by the Sports Lottery Fund, and purchased by village committees, significantly increased participation. The presence of social sports instructors also had a significant impact on increasing the time and frequency of residents’ sports activities, emphasizing the importance of optimizing the allocation of sports facilities and guidance resources.

The reasons for this phenomenon may be that increasing age is often accompanied by a decline in physical ability and an increase in health problems (18, 19), which may limit the ability of rural residents to participate in high-intensity or long-duration sports activities.Among the 20.45% of individuals who did not participate in sports activities, a significant proportion were older adults (aged 60 and above), likely restricted by declining physical capacity and health issues. Low-income groups (annual income below 10,000 RMB) and those with lower education levels (middle school or below) were also overrepresented, reflecting the impact of economic pressures and limited health awareness. Additionally, underdeveloped regions such as the northeast and west faced further constraints due to insufficient accessibility to sports facilities. These findings suggest the need to design suitable activities for older adults, provide economic support for low-income groups, and prioritize infrastructure development in underdeveloped areas to reduce barriers to participation. Additionally, as age increases, the social roles and responsibilities of villagers may change, such as increased family and work pressures, which can reduce the free time available for sports activities (20, 21). Individuals with higher education levels may be more engaged in mental rather than physical labor and may spend more time on indoor activities due to the nature of their work, thereby reducing opportunities to participate in sports activities.

The positive correlation between occupation and household income suggests that families with better economic conditions may have more resources and opportunities to participate in sports activities (22), such as purchasing gym memberships, attending fitness classes, or paying for sports-related expenses. Moreover, higher income levels may be associated with occupations that offer more free time and higher quality of life, thereby increasing the likelihood of participating in sports activities. The negative correlation between sports activity time and age and education, and the positive correlation with occupation and household income (23), reflect the combined effects of individuals’ physical conditions, social responsibilities, economic resources, and time allocation.

### 4.2 The impact of sources of public sports facilities on rural residents’ participation in sports activities

This study found a significant correlation between the sources of public sports facilities and the level of sports activity participation among rural residents, including the positive promotion effects of facilities donated by the Sports Lottery Fund, provided by township governments, and purchased by village committees.

The availability of public sports facilities can be seen as a form of social capital that fosters interaction and social cohesion among community members (24, 25). The investment by township governments and village committees in providing sports facilities not only enhances the accessibility of sports activities but also reflects a focus on public health and well-being, thereby strengthening residents’ sense of social belonging and community involvement, which promotes active participation in sports activities among rural residents. The quality and quantity of public sports facilities directly influence the level of participation in sports activities. High-quality sports facilities can provide a safer and more comfortable environment for exercise, attracting rural residents to participate actively. Moreover, the diversity of sports facilities allows residents of different ages, genders, and physical conditions to find suitable sports activities, thereby improving the overall level of sports participation (26).

Economic incentives play a crucial role in promoting participation in sports activities (27, 28). Donations from the Sports Lottery Fund and other forms of financial support significantly reduce the economic burden of constructing and maintaining sports facilities in rural areas, making sports activities more affordable for ordinary residents. One of the core theories of economics is that people make rational choices between marginal costs and marginal benefits; when the economic cost of sports activities decreases, the marginal benefits of participating in sports activities relative to the marginal costs increase (29), thereby enhancing their willingness to participate. Additionally, the injection of economic resources not only improves existing sports facilities but also introduces more sports programs and activities, increasing the diversity of choices for residents and further stimulating their enthusiasm for participation.

The availability and accessibility of public sports facilities are crucial for enhancing individuals’ intentions and motivations to engage in sports activities (30–32). The presence of public sports facilities can inspire a sense of psychological ownership among individuals, meaning a sense of belonging to community sports facilities (33), which can increase rural residents’ community involvement and intrinsic motivation for sports activities. At the same time, government and community support for sports activities manifests not only in the provision of material resources but also in positive reinforcement of residents’ sports activities (34), which is an important psychological resource for increasing individuals’ motivation to participate. The social modeling effect, wherein observing others (especially those nearby) engaging in sports activities, can encourage more residents to emulate and join sports activities through social learning mechanisms. Cultural customs and community traditions significantly influence trends in sports participation. In some rural areas, the perception of sports activities as purely recreational may hinder participation among women and older adults, while collective activities (e.g., square dancing and traditional martial arts) are more appealing due to their communal nature. Future research should focus on the impact of cultural practices and social expectations on participation behaviors and design culturally tailored interventions to enhance the effectiveness of such activities. This is also a powerful mechanism for promoting sports participation among rural residents. Social learning theory suggests that individuals acquire behaviors not only through direct experience but also by observing and imitating others’ behaviors. In the context of rural Chinese communities, when rural residents see their neighbors, friends, or family members actively participating in sports activities, these behaviors serve as positive social models, stimulating the desire of other community members to imitate.

The significant positive correlation between the sources of public sports facilities and the level of sports activity participation among rural residents is the result of multiple intertwined factors (35, 36). Sociology provides the perspectives of social capital and community participation; sports science emphasizes the impact of facility quality on sports participation (37); economics explains the role of financial support in the accessibility and affordability of facilities (38); and psychology highlights the impact of facility availability on individual motivation and behavior. Therefore, enhancing the participation of rural residents in sports activities requires the concerted efforts of the government, community, and individuals in terms of resource investment, facility construction, and psychological support.

### 4.3 The impact of public social sports instructors on rural residents’ participation in sports activities

Sports facility instructors in rural communities play multiple roles that extend beyond technical guidance. They are also catalysts for social interaction and community cohesion. By organizing and guiding sports activities, instructors provide a platform for community members to gather and participate collectively (39), enhancing connections and interactions among rural residents and promoting the establishment of social cohesion. Urban residents working in villages and retired urban residents who return to the countryside, with their rich life experiences and possible social status, become respected and trusted role models within the community. Their participation not only increases the social recognition of sports activities but also motivates more residents to emulate their behavior and participate in sports activities, thereby creating a positive social modeling effect.

Through the involvement of public sports facility instructors, rural sports can be effectively managed and operated. Instructors can significantly reduce the marginal costs of residents’ participation in sports activities by organizing free or low-cost sports activities and providing easily accessible sports resources, thereby encouraging broader community participation by lowering economic barriers. Additionally, greater group participation in sports activities attracts funding support from the government and institutions, such as donations from the Sports Lottery Fund, providing an important financial foundation for the construction and maintenance of sports facilities, making sports activities a service open to all community members, not just those with better economic conditions.

Professional guidance and high-quality sports facilities are crucial for increasing residents’ participation in sports activities (40). Instructors can provide technical guidance, help residents improve their sports skills, use public sports facilities properly, engage in sports activities safely to reduce injury risk, and design attractive sports programs and activities to meet the needs of residents of different ages, genders, and ability levels. This personalized and inclusive sports service increases residents’ willingness to participate and enhances the popularity of sports activities.

In this study, social sports instructors play a critical role in enhancing rural residents’ participation in sports activities by acting as both technical guides and social behavior regulators. These instructors influence participants’ motivation through various mechanisms, including providing social support, modeling positive behavior, and fostering a sense of community belonging. The findings indicate that the presence of social sports instructors significantly increases the time and frequency of participation (B = 0.265, *p* < 0.001), while their absence leads to a noticeable decline in activity levels (B = -0.445, *p* < 0.001). Furthermore, retired urban residents serving as instructors exerted a particularly strong influence, leveraging their life experiences and authority to inspire greater participation. This highlights the instructors’ ability to shape social norms around regular sports engagement and motivate individuals across diverse age groups, genders, and ability levels. These findings underscore the dual function of social sports instructors in promoting both technical competence and psychosocial engagement, which collectively enhance the overall effectiveness of rural sports programs. Future research could further explore these behavioral mechanisms by measuring specific aspects of social support and norm-setting, as well as integrating motivational theories such as self-determination theory to better capture the underlying motivational processes.

The government and related institutions play a decisive role in providing and maintaining sports facilities and equipping them with professional instructors (41). Policy support and investment are key to establishing and maintaining sports facility infrastructure, and the training and deployment of instructors directly affect the quality and effectiveness of sports activities (42, 43). By formulating and implementing strategies and policies aimed at increasing rural residents’ participation in sports activities, the government can effectively promote the development of community sports activities, improving residents’ health levels and quality of life.

Therefore, sports facility instructors play a crucial role in rural communities, providing not only technical guidance but also a comprehensive impact on social interaction, economic incentives, policy promotion, and increased participation in sports activities. Together, they promote the increase in time and frequency of rural residents’ participation in sports activities.

### 4.4 Summary

This study explored the sports activities of rural residents through sociology, economics, psychology, and sports science. It emphasized the role of social capital and community cohesion in promoting rural residents’ participation in sports activities, pointing out that public sports facilities, as a form of social capital, can promote interaction and social cohesion among community members. Similarly, sports facility instructors in rural sports perform multiple functions, including technical guidance, social interaction, and policy promotion.

Policymakers and rural community organizers need to consider providing customized sports activity programs for different age groups, educational backgrounds, and economic conditions to promote wider social group participation in sports activities and improve overall health levels and quality of life. Increasing investment and maintenance of public sports facilities, through government distribution and organizational donations, helps reduce the economic threshold for sports activities, promoting the popularity and affordability of sports facilities. Fully leveraging the role of social sports instructors can provide not only professional technical guidance but also promote community interaction and cohesion. The government and community should strengthen the training and support of public sports facility instructors, encourage urban residents to serve as rural social sports instructors, and actively participate in the organization and management of sports activities, thereby increasing the willingness and frequency of rural residents’ participation in sports activities.

### 4.5 Limitations of this study

1. The selection criteria for participants in this study included residents aged 18 and above living in specific areas, excluding individuals with health or cognitive issues. This study did not include children, which may limit the generalizability of the results. The exclusion of this demographic may result in findings that do not fully capture the sports participation patterns of the rural population.

These selection criteria may lead to sample limitations, such as excluding those with poor health conditions from completing the questionnaire, making it difficult to generalize the results to all rural residents.

1. This study used self-reported questionnaires to evaluate the level of participation in sports activities, which may be subject to recall bias and subjective evaluation issues. This study did not fully account for the influence of cultural factors on sports participation. Cultural perceptions and social norms across different regions or groups may significantly affect participation levels. Future research could employ qualitative methods, such as interviews or focus groups, to more comprehensively explore the role of cultural and social dynamics in shaping participation behaviors. Respondents’ answers may be influenced by memory, social desirability, and self-perception, potentially leading to overestimation or underestimation of their participation in sports activities, thereby affecting the reliability and accuracy of the data. Future research could consider combining activity trackers or performance assessments to improve the objective evaluation of participation levels in sports activities.
2. Although this study adopted a segmented sampling method to ensure broad representativeness of the sample, there are still some limitations. The selection of samples may be affected by geographical, economic and cultural factors and may not fully cover the diversity of rural residents in China.

## 5 Conclusion

This study conducted an in-depth investigation into the impact of public sports facilities and their instructors on the participation of rural residents in sports activities through the analysis of 3,956 valid questionnaires. The study found that sports activity participation is negatively correlated with age and education level and positively correlated with household income. The sources of public sports facilities significantly influence the promotion of sports activities among rural residents, especially facilities provided by county sports bureaus, donated by the Sports Lottery Fund, and community donations, which significantly increased the frequency of residents’ sports activities. The presence of sports facility instructors plays an important role in the time and frequency of residents’ participation in sports activities. Urban residents working in rural areas and retired urban residents returning to the countryside have made positive contributions to increasing sports activity participation. Policymakers need to emphasize the construction and management of public sports facilities, focus on developing and utilizing diversified funding sources, and enhance the role of sports facility instructors. By providing professional guidance and organizing a variety of sports activities, it is possible to effectively stimulate the enthusiasm of rural residents for participation. Efforts should be made to enhance the construction and management of public sports facilities in underdeveloped regions, reduce participation barriers for low-income groups, and fully leverage the role of instructors in organizing diverse activities. Future studies could adopt longitudinal designs to evaluate the long-term impact of facilities and instructors on participation levels and health outcomes, providing a foundation for more sustainable policymaking.

## Data Availability

The raw data supporting the conclusions of this article will be made available by the authors, without undue reservation.
